# Mesoporous‐Shell Monolayer Plasmonic Architecture Enables Quantitative and Decision‐Guided SERS

**DOI:** 10.1002/advs.77299

**Published:** 2026-08-24

**Authors:** Guangyao Huang, Ying Wang, Yue Fang, Jingyuan Shao, Sensen Hao, Mancun Zhao, Yuan Yao, Junping Wang, Wei Zhou, Hongzhi Wang

**Affiliations:** ^1^ Hefei Cancer Hospital of CAS Institute of Health and Medical Technology Hefei Institutes of Physical Science Chinese Academy of Sciences (CAS) Hefei Anhui China; ^2^ University of Science and Technology of China Hefei China; ^3^ Beijing Institute of Radiation Medicine Beijing China

**Keywords:** decision‐guided sensing, deterministic hotspots, mesoporous‐shell monolayer architecture, plasmonic nanostructures, quantitative SERS

## Abstract

Quantitative surface‐enhanced Raman spectroscopy (SERS) has long been impeded by stochastic hotspot formation, signal instability, and limited chemical fidelity in complex environments. Overcoming these intrinsic limitations requires structural control at the nanoscale rather than incremental improvements in signal amplification. Here, we introduce a mesoporous‐shell monolayer plasmonic architecture that enables quantitative and decision‐guided SERS by integrating deterministic hotspot geometry with regulated molecular accessibility and chemical stabilization of photosensitive analytes. This architecture produces predictable and reproducible SERS responses, shifting SERS from stochastic enhancement toward quantitative sensing. Using chemotherapeutic drugs as representative small‐molecule targets, the platform demonstrates ultrasensitive, multiplexed, and reproducible detection in standard solutions and complex biological matrices. Machine learning–assisted spectral analysis further enables accurate concentration prediction in patient blood samples in close agreement with liquid chromatography–mass spectrometry. Importantly, SERS‐guided dose adjustment in an orthotopic breast cancer mouse model maintains therapeutic efficacy while significantly reducing systemic toxicity, establishing a direct link between plasmonic sensing and decision‐guided intervention. This work defines a materials‐based framework for quantitative and decision‐enabled SERS, in which mesoporous‐shell monolayer architectures transform plasmonic nanostructures into functional platforms for real‐world molecular quantification and feedback‐controlled biomedical applications.

## 1 Introduction

Chemotherapy remains a cornerstone of systemic cancer treatment; however, its effectiveness is fundamentally constrained by the lack of quantitative control over drug exposure rather than by drug availability [1, 2]. Many widely used chemotherapeutic agents, including alkylating agents and anthracyclines, exhibit narrow therapeutic windows, where insufficient systemic exposure compromises tumor suppression while excessive exposure leads to severe dose‐limiting toxicities [3, 4, 5]. Despite advances in precision oncology, dose determination in routine chemotherapy still relies predominantly on population‐based parameters such as body surface area or fixed regimens, rather than real‐time molecular‐level measurements. Such empirical strategies fail to account for pronounced interindividual variability in pharmacokinetics, resulting in substantial discrepancies in blood drug exposure among patients receiving identical doses [6]. As a result, heterogeneous therapeutic outcomes and unpredictable adverse effects persist, underscoring the need for quantitative and individualized drug monitoring strategies.

Quantitative measurement of drug concentrations in biological fluids has long been proposed as a rational strategy to enable individualized dose adjustment [7, 8]. Such measurements directly reflect individual pharmacokinetic behavior and provide a pathway toward controllable and personalized chemotherapy. Liquid chromatography–mass spectrometry (LC–MS) is widely regarded as the gold standard for Therapeutic Drug Monitoring (TDM) owing to its excellent sensitivity and molecular specificity [9]. However, recent perspectives increasingly emphasize that next‐generation quantitative drug monitoring requires not only analytical accuracy but also rapid turnaround time and compatibility with frequent measurements. In this regard, LC–MS is intrinsically constrained by labor‐intensive sample preparation, sophisticated instrumentation, and centralized laboratory workflows, which collectively limit temporal resolution and accessibility. These constraints hinder near‐real‐time or high‐frequency molecular monitoring, where timely feedback is essential for dose optimization.

Surface‐enhanced Raman spectroscopy (SERS) has emerged as a promising alternative analytical modality due to its label‐free detection, molecular fingerprint specificity, and potential for rapid analysis with minimal sample consumption [10]. In principle, SERS could enable direct molecular quantification in complex biological matrices, aligning with emerging demands for decentralized and high‐frequency chemical sensing [11]. Accordingly, recent research trends in SERS have shifted from proof‐of‐concept demonstrations toward biofluid analysis under clinically realistic constraints. Nevertheless, despite extensive advances in plasmonic nanostructure engineering, the realization of quantitative and reliable SERS in complex biological environments remains elusive. Three fundamental barriers persist: instability of SERS signals in complex biological environments, poor reproducibility caused by stochastic hotspot distributions, and the intrinsic difficulty of achieving reliable quantitative analysis rather than qualitative or semi‐quantitative detection. These challenges have largely confined SERS to simplified analytical conditions, falling short of the robustness required for quantitative sensing in physiologically complex systems [12].

Beyond these widely acknowledged limitations, recent methodological studies have highlighted an additional and often underappreciated failure mode in quantitative SERS pipelines: chemical instability of analytes during optical interrogation [13]. This issue is particularly relevant for clinically important photosensitive drugs, such as anthracyclines including epirubicin (EPI), which are prone to laser‐induced degradation [14]. When directly exposed to plasmonic metal surfaces under laser illumination, such molecules may undergo photochemical reactions that alter their molecular structure and Raman fingerprints over time. The resulting spectral drift and signal decay fundamentally undermine quantitative reliability, particularly in applications that demand chemically faithful and temporally stable molecular readouts. Despite its importance, photochemical instability has rarely been systematically addressed through nanostructural design principles in SERS substrates.

Collectively, these considerations underscore a critical conceptual shift required for quantitative SERS in complex biological environments. Unlike conventional SERS designs that prioritize maximal electromagnetic enhancement, quantitative and decision‐guided sensing demands nanostructures optimized for robustness, predictability, and chemical stability in complex environments [15, 16]. Addressing these requirements cannot be achieved solely through algorithmic correction or post hoc calibration; rather, it necessitates a synergistic nanostructural design that integrates controlled molecular accessibility, suppression of biological interference, protection of analyte integrity under laser irradiation, and deterministic hotspot geometry. Recent analyses increasingly identify nanostructure‐level solutions as the decisive factor in overcoming the quantitative and translational bottlenecks of SERS‐based molecular sensing [11, 12].

**SCHEME 1:**
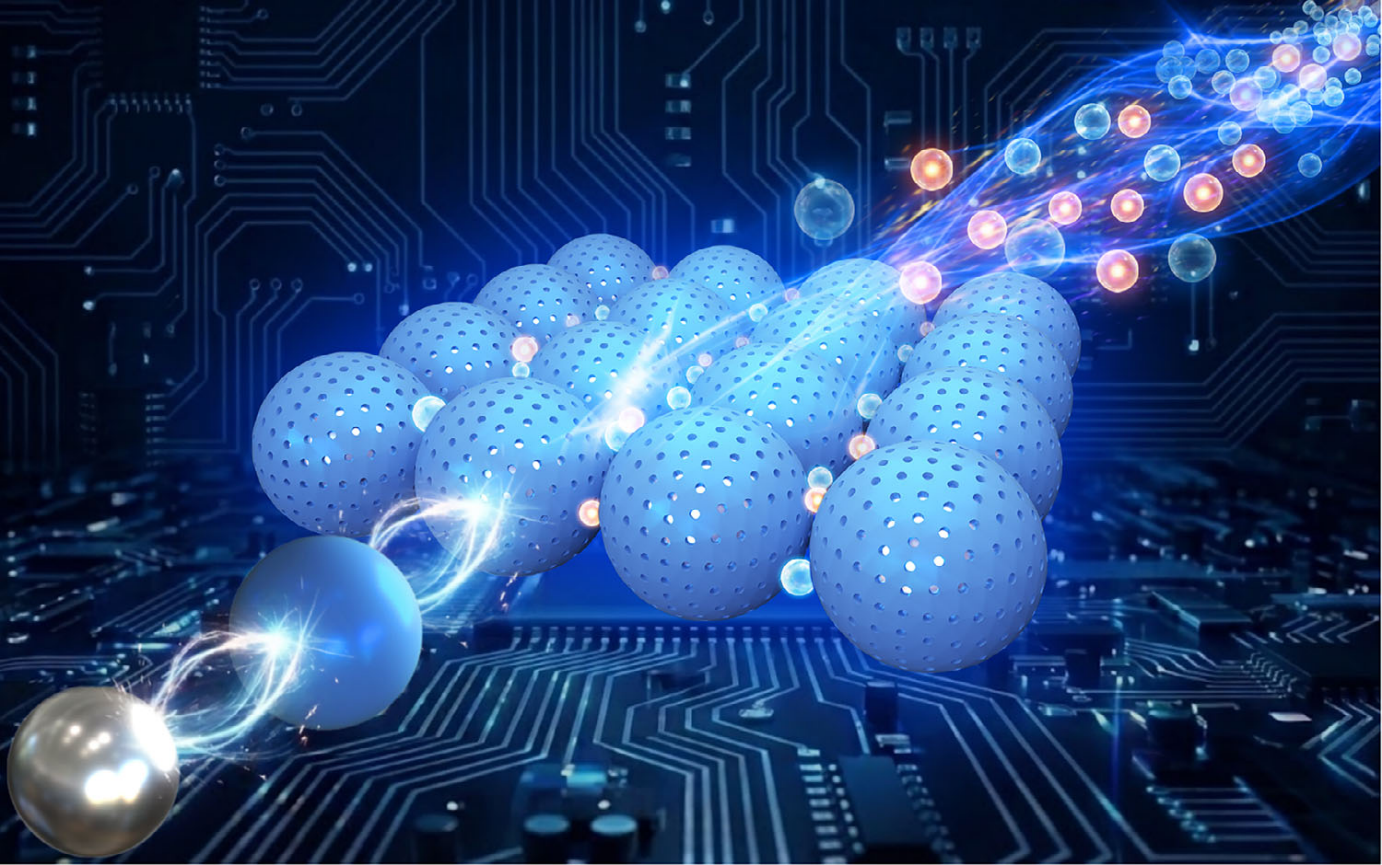
Mesoporous‐shell‐regulated molecular transport combined with monolayer hotspot determinism transforms surface‐enhanced Raman spectroscopy (SERS) from ultrasensitive detection into quantitative and decision‐guided molecular sensing.

Here, we report a mesoporous SiO_2_ shell–enabled monolayer plasmonic architecture specifically designed to meet the emerging requirements of quantitative, matrix‐robust, and decision‐guided SERS. By coating silver nanoparticles with a mesoporous SiO_2_ shell and assembling them into a controlled monolayer architecture, this platform enables selective diffusion of small‐molecule chemotherapeutic drugs while excluding macromolecular interferents, mitigates laser‐induced photodegradation of light‐sensitive agents such as epirubicin through spatial confinement and chemical shielding, and stabilizes local electromagnetic environments in complex biological media. The monolayer configuration further ensures deterministic hotspot distribution, suppressing stochastic signal amplification and enabling reproducible, predictable SERS responses suitable for quantitative analysis (Scheme 1). Using this architecture as a proof‐of‐concept, we demonstrate quantitative and multiplexed detection of representative small‐molecule drugs in serum, accurate concentration prediction validated against LC–MS in patient blood samples via machine learning–assisted analysis, and decision‐guided intervention in an orthotopic breast cancer mouse model that reduces systemic toxicity without compromising therapeutic efficacy. Collectively, this work advances SERS from a stochastic enhancement technique to a decision‐enabling, architecture‐driven nanotechnology for quantitative molecular sensing and feedback‐controlled biomedical applications.

## 2 Results and Discussion

### 2.1 Mesoporous‐Shell Monolayer Plasmonic Architecture for Deterministic Hotspot Formation

**FIGURE 1:**
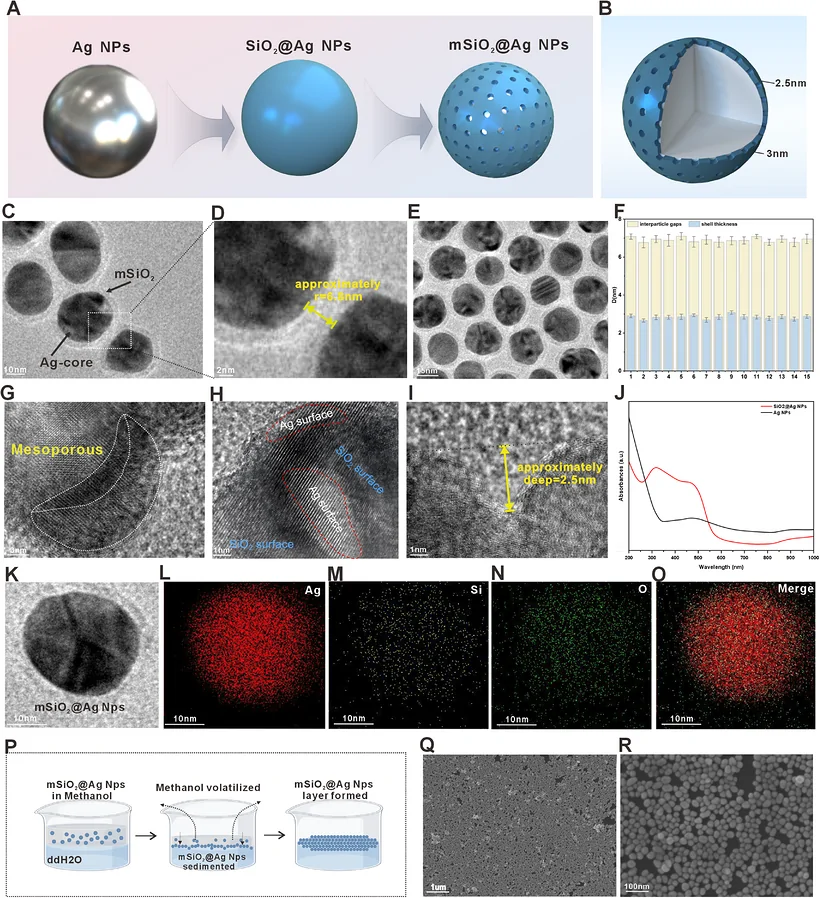
Fabrication and Characterization of Monolayer SERS Substrates Composed of mSiO2@Ag NPs. (A) Construction process of mSiO2@Ag NPs. (B) Detailed dimensions of the particles. (C) Transmission electron microscopy (TEM) image of mSiO_2_@Ag NPs. (D) The TEM image of mSiO_2_@Ag NPs’ gap. (E) TEM image of the monolayer assembled mSiO_2_@Ag NP substrate. (F) Statistical analysis of shell thickness and interparticle distance. (G) Mesoporous morphology captured by TEM. (H) Schematic representation of the mesoporous SiO_2_‐coated Ag nanoparticle and an enlarged HRTEM image of the Ag‐SiO_2_ interface. (J) Comparison of UV–vis absorbance between mSiO_2_@Ag NPs and Ag NPs. (K) Morphology TEM image of mSiO_2_@Ag NPs. (L–N). Elemental mappings of Ag, Si, and O for mSiO_2_@Ag NPs. (O) Merged image of Ag, Si, and O elements for mSiO_2_@Ag NPs. (P) Schematic diagram of the air–liquid interface self‐assembly process. (Q, R) Scanning electron microscopy (SEM) images of monolayer substrates formed by particle self‐assembly.

To achieve quantitative and structurally predictable surface‐enhanced Raman scattering (SERS), mesoporous silica‐coated silver nanoparticles (mSiO_2_@Ag NPs) were rationally designed and assembled into an ordered monolayer plasmonic architecture [17, 18]. As illustrated in Figure 1A,B, the fabrication process involves sequential formation of Ag cores, deposition of a dense SiO_2_ shell, and subsequent generation of mesoporous structures, yielding nanoparticles with well‐defined core–shell geometry and tunable structural parameters. Transmission electron microscopy (TEM) images reveal that the Ag nanoparticles exhibit high morphological uniformity and are successfully encapsulated by a homogeneous mesoporous SiO_2_ shell (Figure 1C–E and Figure S1). Statistical analysis indicates that the SiO_2_ shell thickness is approximately 2.5 nm, while the interparticle spacing is around 6.8 nm (Figure 1F–I). TEM imaging (Figure 1H) further clarifies the mesoporous structure at the Ag–SiO_2_ interface. Under identical imaging conditions, regions where the Ag (111) lattice fringes are clearly resolved are assigned to Ag surfaces exposed through pore openings, whereas regions where the Ag lattice fringes are obscured are assigned to Ag surfaces covered by SiO_2_, corresponding to non‐pore regions. This well‐defined mesoporous shell establishes nanoscale mass‐transport channels that enable selective diffusion of small‐molecule analytes while effectively excluding macromolecular interferents, thereby enhancing both selectivity and matrix tolerance at the structural level.

Notably, the interparticle gap of ≈6.8 nm falls within the optimal regime for electromagnetic hotspot formation (Figure 1D,F), supporting stable and reproducible plasmonic coupling while avoiding excessive field amplification associated with random nanoparticle aggregation. Compared with bare Ag nanoparticles, the mSiO_2_@Ag NPs exhibit a pronounced redshift and spectral broadening in the UV–vis absorption spectrum (Figure 1J), indicating that the mesoporous shell modifies the local dielectric environment and induces enhanced plasmonic coupling.

High‐resolution TEM and elemental mapping further confirm the uniform coexistence of Ag and Si elements (Figure 1K–O), verifying the successful construction of the core–shell structure. To translate these nanoscale building blocks into a macroscopic functional interface, an air–liquid interfacial self‐assembly strategy was employed (Figure 1P). Scanning electron microscopy (SEM) images demonstrate the formation of large‐area, highly uniform monolayer films (Figure 1Q,R) [19, 20]. This monolayer configuration effectively suppresses vertical stacking of nanoparticles and converts stochastic hotspot distributions into structurally deterministic ones, thereby significantly improving signal reproducibility and predictability.

Collectively, the synergistic integration of mesoporous shell engineering and ordered monolayer assembly endows the SERS substrate with controlled molecular accessibility, reduced background interference, and reproducible plasmonic coupling. Rather than maximizing signal enhancement, this design prioritizes structural determinism and quantitative reliability, which are essential prerequisites for SERS analysis in complex biological systems. Distinct from conventional randomly aggregated substrates, this architecture provides a robust structural foundation for subsequent quantitative molecular sensing and intelligent analytical applications.

### 2.2 Deterministic Hotspot Engineering Enables Quantitative and Matrix‐Robust SERS

**FIGURE 2:**
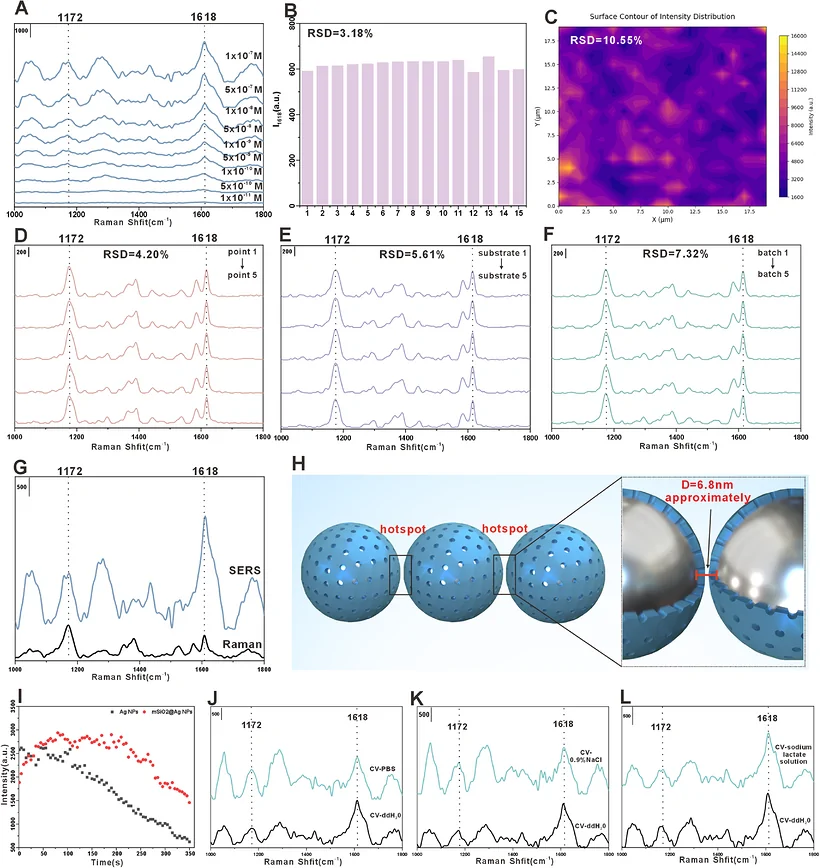
Verification of detection performance and mechanism investigation of the mSiO_2_@Ag NPs self‐assembled substrate. (A) Detection of CV aqueous solutions at different concentrations using the mSiO_2_@Ag NPs self‐assembled substrate. (B) The relative standard deviation (RSD) of 15 repeated detections of 5 × 10^−10 ^M CV using the mSiO_2_@Ag NPs self‐assembled substrate. (C) Heat map of Raman mapping results from scanning 100 points on the substrate for CV detection. (D–F) Consistency evaluation across multiple points, substrates, and batches. (G) Comparison of peak intensities between SERS and Raman spectroscopy for CV detection and calculation of the enhancement factor. (H) Schematic illustration of the substrate simulation and display of interparticle gaps. (I) Peak intensity vs. time profiles for epirubicin detection using the mSiO_2_@Ag NPs self‐assembled substrate and a conventional Ag NPs substrate. (J) Comparison of detection results for CV in PBS solution vs. ddH_2_O. (K) Comparison of detection results for CV in saline solution vs. ddH_2_O. (L) Comparison of detection results for CV in sodium lactate solution vs. ddH_2_O.

The structure–function relationship of the mesoporous‐shell monolayer architecture was first examined using crystal violet (CV) as a model Raman probe. As shown in Figure 2A, characteristic Raman peaks of CV at 1172 and 1618 cm^−1^ were clearly resolved over a broad concentration range down to 5 × 10^−10^ M, indicating the ultrasensitive response enabled by the monolayer plasmonic architecture. Importantly, repeated measurements at this ultralow concentration exhibited excellent signal stability, with a relative standard deviation (RSD) of only 3.10% over 15 independent detections (Figure 2B and Figure S2), supporting the quantitative reliability of the architecture. Raman mapping over 100 randomly selected points revealed a highly uniform intensity distribution, and repeated tests among multiple points, multiple substrates, and multiple batches confirmed the large‐area structural homogeneity of the monolayer assembly and the reproducibility of deterministic hotspot formation (RSD = 10.55%, 4.20%, 5.61%, and 7.32%) (Figure 2C–F and Figures S3–S5). A stable linear correlation between CV concentration and SERS peak intensity was observed over the tested range (Figure S6), further demonstrating that the architecture supports quantitative rather than merely qualitative signal transduction.

To elucidate the origin of this robust response, the SERS spectra were compared with conventional Raman measurements of CV (Figure 2G), revealing a pronounced enhancement arising from plasmonic coupling between adjacent nanoparticles (enhancement factor = 5.16 × 10^7^). The schematic of the assembled structure highlights the presence of interparticle gaps (∼6.8 nm), which act as deterministic electromagnetic hotspots (Figure 2H). Unlike random nanoparticle aggregates that generate sporadic and extreme field amplification, the monolayer configuration enforces predictable hotspot geometry and suppresses stochastic signal fluctuations, thereby favoring quantitative rather than merely ultrasensitive detection. Notably, compared with bare Ag NPs substrates, the mesoporous‐shell architecture exhibited markedly improved time‐dependent signal photostability for epirubicin (5 × 10^−9^ M) under continuous laser irradiation, indicating effective suppression of laser‐induced photodegradation (Figure 2I).

Beyond sensitivity and reproducibility, the architecture exhibited pronounced robustness in complex ionic environments that mimic physiologically relevant conditions. As shown in Figure 2J–L, the SERS responses of CV in phosphate‐buffered saline (PBS), saline, and sodium lactate solutions closely resembled those obtained in double‐distilled water (ddH2O), with minimal signal attenuation or spectral distortion. This robustness against ionic strength and metabolite interference can be attributed to the mesoporous SiO_2_ shell, which regulates molecular diffusion and reduces nonspecific adsorption while stabilizing the local electromagnetic environment. Together, these results demonstrate that the mesoporous‐shell monolayer architecture integrates deterministic hotspot geometry with chemical shielding, enabling stable, uniform, and matrix‐resilient SERS—key prerequisites for quantitative and decision‐guided molecular sensing in complex environments.

### 2.3 Architecture‐Driven Quantification and Multiplexed Spectral Discrimination

**FIGURE 3:**
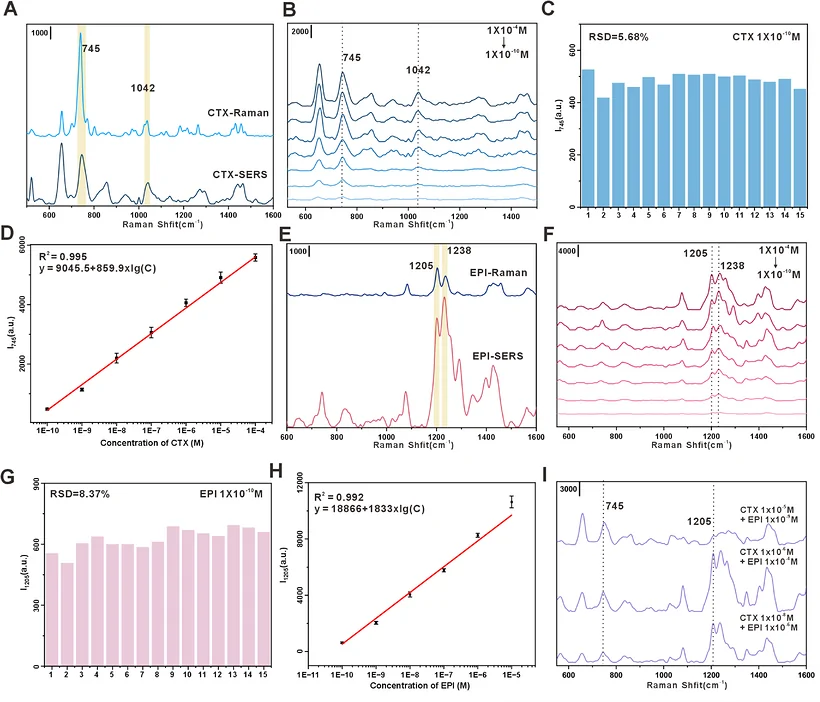
Detection of Standard solutions of CTX and EPI by the mSiO2@Ag NPs self‐assembled substrate. (A) Comparison of characteristic peaks between SERS and Raman spectra of CTX (Raman: CTX crystals (≈100% purity); SERS: CTX‐ddH2O solution (1 × 10^−7 ^M). (B) Detection of CTX at different concentrations using the mSiO2@Ag NPs self‐assembled substrate. (C) The RSD from 15 consecutive measurements of CTX (1 × 10^−10^ M). (D) Concentration‐peak intensity calibration curve for CTX. (E) Comparison of characteristic peaks between SERS and Raman spectra of EPI (Raman: EPI crystals (≈100% purity); SERS: EPI‐ddH2O solution (1 × 10^−7 ^M). (F) Detection of EPI at different concentrations using the mSiO2@Ag NPs self‐assembled substrate. (G) The RSD from 15 consecutive measurements of EPI (1 × 10^−10^ M). (H) Concentration‐peak intensity calibration curve for EPI. (I) Detection of mixed‐standard solutions of CTX and EPI by the mSiO2@Ag NPs self‐assembled substrate at different concentrations.

The architecture‐driven quantitative sensing capability was evaluated using cyclophosphamide (CTX) and epirubicin (EPI) as representative small‐molecule analytes. Distinct characteristic Raman bands of CTX were clearly resolved in the SERS spectra and exhibited excellent agreement with those obtained from conventional Raman measurements (Figure 3A), confirming the chemical fidelity of the SERS fingerprints. Upon decreasing the CTX concentration, the characteristic peaks remained discernible down to 1 × 10^−10^ M (Figure 3B), indicating the ultrasensitive response enabled by the mesoporous‐shell monolayer architecture. Repeated measurements at this ultralow concentration yielded an RSD of 5.68% over 15 consecutive acquisitions (Figure 3C), demonstrating the reproducibility and quantitative stability of the architecture.

A linear relationship between the SERS peak intensity and CTX concentration was established over the tested range (Figure 3D), indicating reliable quantitative transduction rather than merely qualitative detection. A similar quantitative response was observed for EPI. The SERS spectra of EPI retained their characteristic vibrational features when compared with conventional Raman spectra (Figure 3E), and concentration‐dependent signal evolution was observed down to 1 × 10^−10^ M (Figure 3F). The architecture exhibited excellent measurement consistency for EPI with an RSD of 4.87% at this low concentration (Figure 3G), and a strong linear correlation between peak intensity and concentration was achieved (Figure 3H). The calculated limit of detection (LOD) and limit of quantification (LOQ) for EPI detection are 3.95 × 10^−10^ and 1.32 × 10^−9^ M, respectively, while those for CTX are 1.18 × 10^−10^ and 3.93 × 10^−10^ M, which satisfactorily meet the requirements for clinical detection. Consistent signal reproducibility was also observed at higher analyte concentrations, further demonstrating the robustness of the monolayer architecture across a broad dynamic range (Figure 2I). These results collectively demonstrate that the architecture supports robust and reproducible quantification of small‐molecule analytes with distinct molecular structures. To further demonstrate the generality of this architecture, additional small‐molecule analytes including carboplatin and dacarbazine were also successfully detected, exhibiting well‐preserved Raman fingerprints and concentration‐dependent responses (Figures S7 and S8).

Beyond single‐analyte sensing, the ability of the architecture to discriminate mixed molecular systems was further examined using CTX and EPI in combination. As shown in Figure 3I, characteristic Raman peaks corresponding to both drugs could be simultaneously identified in mixed‐standard solutions across different concentration ratios, indicating minimal spectral cross‐interference and preserved molecular specificity. Such multiplexed spectral discrimination is critical for quantitative sensing in multicomponent molecular systems. The successful discrimination and quantification of CTX and EPI in mixed solutions underscore the potential of the mesoporous‐shell monolayer architecture as a generalizable platform for multiplexed quantitative SERS.

Taken together, these results establish that the rationally engineered mesoporous‐shell monolayer architecture enables not only ultrasensitive but also quantitatively reliable and multiplexed molecular detection. The combination of deterministic hotspot geometry and mesoporous‐shell–mediated chemical stabilization ensures that the observed SERS responses faithfully reflect molecular concentration rather than stochastic enhancement effects, providing a structural foundation for subsequent investigations of quantitative SERS in complex environments.

### 2.4 Mesoporous‐Shell–Regulated Molecular Transport for SERS in Complex Biological Media

**FIGURE 4:**
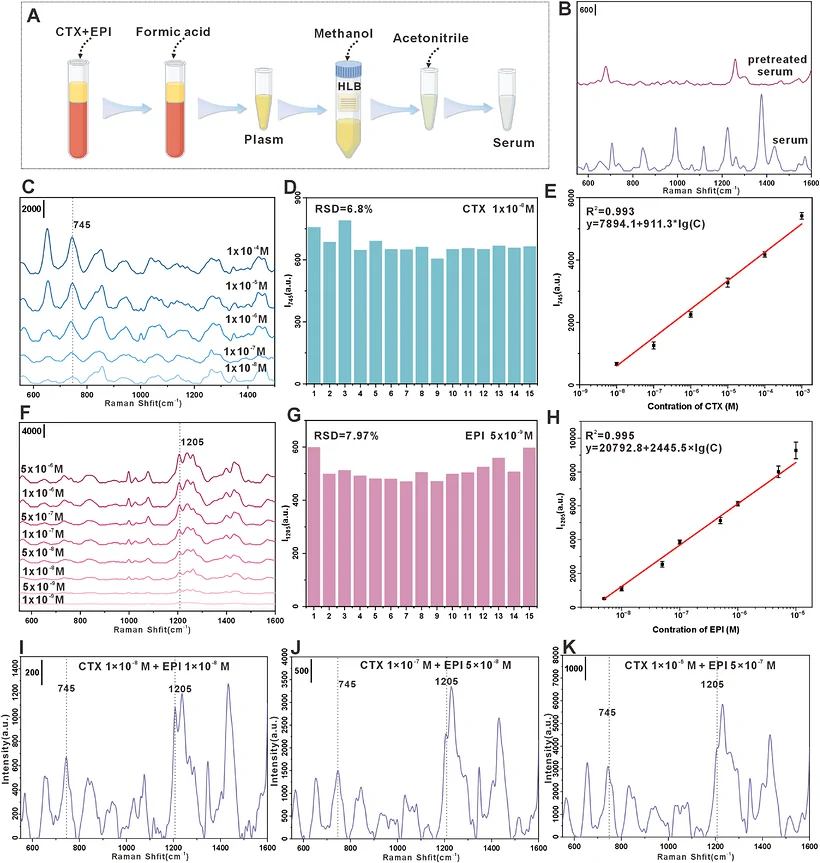
Detection of CTX and EPI in serum solution using the mSiO_2_@Ag NPs self‐assembled substrate. (A) Schematic diagram of the serum pretreatment process. (B) SERS spectra of serum before and after pretreatment. (C) Detection of CTX‐serum solutions at different concentrations using the mSiO_2_@Ag NPs self‐assembled substrate. (D) 15 consecutive detections of CTX‐spiked serum solution (1 × 10^−8^ M) using the mSiO_2_@Ag NPs self‐assembled substrate. (E) Concentration‐peak intensity calibration curve for CTX‐serum solution. (F) Detection of EPI‐serum solutions at different concentrations using the mSiO_2_@Ag NPs self‐assembled substrate. (G) 15 consecutive detections of EPI‐serum solution (5 × 10^−9 ^M) using the mSiO_2_@Ag NPs self‐assembled substrate. (H) Concentration‐peak intensity calibration curve for EPI in serum solution. (I–K) SERS spectra of serum solutions spiked with CTX+EPI mixtures at different concentrations (CTX 1 × 10^−8^ M+EPI 1 × 10^−8 ^M, CTX 1 × 10^−7^ M+EPI 5 × 10^−8 ^M, CTX 1 × 10^−5^ M+EPI 1 × 10^−7 ^M).

To examine the matrix robustness enabled by mesoporous‐shell–regulated molecular transport, CTX and EPI were used as representative small‐molecule analytes in spiked serum samples following a simplified pretreatment protocol (Figure 4A). As shown in Figure 4B and Figure S9, raw serum exhibited strong background signals that obscured analyte‐related Raman features, whereas after pretreatment, characteristic vibrational peaks became clearly distinguishable, indicating effective removal of macromolecular interferents while preserving small‐molecule species. This preprocessing step enabled reliable SERS interrogation of small‐molecule analytes in physiologically relevant complex media.

The spectral changes in Figure 4B demonstrate that the pretreatment procedure primarily reduces interference from the bulk serum matrix rather than indiscriminately eliminating Raman‐active molecules. Untreated serum contains abundant proteins, lipids, phospholipids, metabolites, and other endogenous constituents that produce broad, overlapping Raman features and can adsorb nonspecifically onto the Ag surface. Prominent serum‐associated features occur in the approximate regions of 700–730, 830–850, around 1000, 1120–1150, 1240–1300, and 1370–1450 cm^−1^. These regions are tentatively associated with nucleobase‐ or phospholipid‐related vibrations, tyrosine‐associated ring vibrations, the phenylalanine ring‐breathing mode, protein‐ or lipid‐associated C─C and C─N stretching vibrations, amide III or lipid CH_2_ modes, and CH_2_/CH_3_ deformation modes, respectively. Because serum is a multicomponent biological matrix, these assignments are approximate and represent overlapping rather than exclusive molecular contributions.

Following acidification, methanol precipitation, and HLB solid‐phase extraction, these protein‐ and lipid‐rich background features are markedly attenuated. Weak residual contributions may remain because of spectral overlap; therefore, these bands are described as attenuated rather than completely absent. The tentative assignments and qualitative changes in band visibility before and after pretreatment are summarized in Table S1.

Importantly, the pretreatment preserves analytically informative drug‐specific Raman features. The CTX marker band at approximately 745 cm^−1^, attributed predominantly to a C─Cl vibration, remains detectable in processed CTX‐containing samples. Similarly, the EPI marker band at approximately 1205 cm^−1^, associated with an anthracycline ring vibration, is retained in processed EPI‐containing samples. These assignments are supported by comparison with the corresponding standard spectra in Figure 3A,E. Their concentration‐dependent responses in Figure 4C,F and simultaneous resolution in the mixed‐analyte spectra in Figure 4I–K further indicate that pretreatment suppresses the serum‐derived background while preserving and unmasking analytically useful drug fingerprints.

Distinct concentration‐dependent SERS responses of CTX in serum were observed over a wide dynamic range (Figure 4C), with characteristic peaks remaining discernible down to 1 × 10^−8^ M, demonstrating effective signal transduction in a complex matrix. Repeated measurements of CTX‐spiked serum at this concentration exhibited excellent signal reproducibility, with a relative standard deviation (RSD) below 10% across 15 consecutive acquisitions (Figure 4D), supporting quantitative reliability in serum matrices. A strong linear correlation between peak intensity and CTX concentration was established (Figure 4E), demonstrating that the architecture supports quantitative SERS even in the presence of complex serum matrices.

A similar quantitative response was observed for EPI. The SERS spectra of EPI in serum retained well‐defined molecular fingerprints across different concentrations (Figure 4F), and repeated measurements at 5 × 10^−9^ M yielded an RSD of approximately 7.97% (Figure 4G). The corresponding calibration curve further confirmed a robust linear relationship between SERS intensity and EPI concentration in serum (Figure 4H), consistent with quantitative molecular transduction.

Importantly, the architecture maintained its molecular specificity under mixed‐analyte conditions. As shown in Figure 4I–K, characteristic Raman peaks of CTX and EPI could be simultaneously resolved in serum solutions containing both analytes at different concentration ratios, without obvious spectral overlap or loss of signal fidelity. Such multiplexed spectral discrimination is essential for quantitative sensing in multicomponent molecular systems. The ability to distinguish and quantify multiple analytes in a single complex biological sample highlights the general applicability of the mesoporous‐shell monolayer architecture for multiplexed quantitative SERS.

The robust performance observed in serum can be attributed to the synergistic effects of mesoporous shell engineering and monolayer hotspot architecture. The mesoporous SiO_2_ shell regulates molecular diffusion and suppresses nonspecific adsorption of macromolecular species, thereby reducing background interference and stabilizing the local electromagnetic environment. Meanwhile, the deterministic hotspot distribution provided by the monolayer assembly minimizes signal fluctuations arising from random nanoparticle aggregation. Together, these features enable chemically faithful and quantitatively reliable SERS in complex biological media, establishing a structural framework for quantitative and decision‐guided molecular sensing in heterogeneous environments.

Figure S9 further separates the contribution of substrate architecture from that of sample pretreatment by comparing identically HLB‐pretreated serum samples measured using substrates assembled from bare Ag, dense SiO_2_@Ag, and mesoporous mSiO_2_@Ag nanoparticles. Bare Ag produces the most complex residual spectrum, consistent with direct and nonspecific adsorption of remaining serum constituents onto the exposed metal surface. Although a dense SiO_2_ shell reduces direct surface contact, its nonporous structure also restricts the diffusion of small analytes toward the enhanced near‐field region. In contrast, the mesoporous SiO_2_ shell limits direct adsorption of larger residual matrix components while retaining transport pathways for smaller molecules. The mesoporous‐shell substrate therefore provides a cleaner spectral background without completely preventing the target molecules from accessing the SERS‐active region.

Taken together, Figure 4B and Figure S9 demonstrate two complementary levels of matrix regulation. Sample pretreatment provides bulk‐matrix purification by removing a substantial proportion of the abundant proteins, lipids, and other endogenous serum constituents, whereas the mesoporous shell regulates molecular transport and nonspecific adsorption at the sensing interface. Thus, the principal advantage of the mesoporous‐shell architecture is not simply an increase in absolute SERS intensity. More importantly, it improves matrix tolerance and spectral fidelity by balancing residual‐matrix exclusion with small‐molecule accessibility.

### 2.5 Machine Learning–Assisted Quantitative SERS for Decision‐Guided Molecular Readout

**FIGURE 5:**
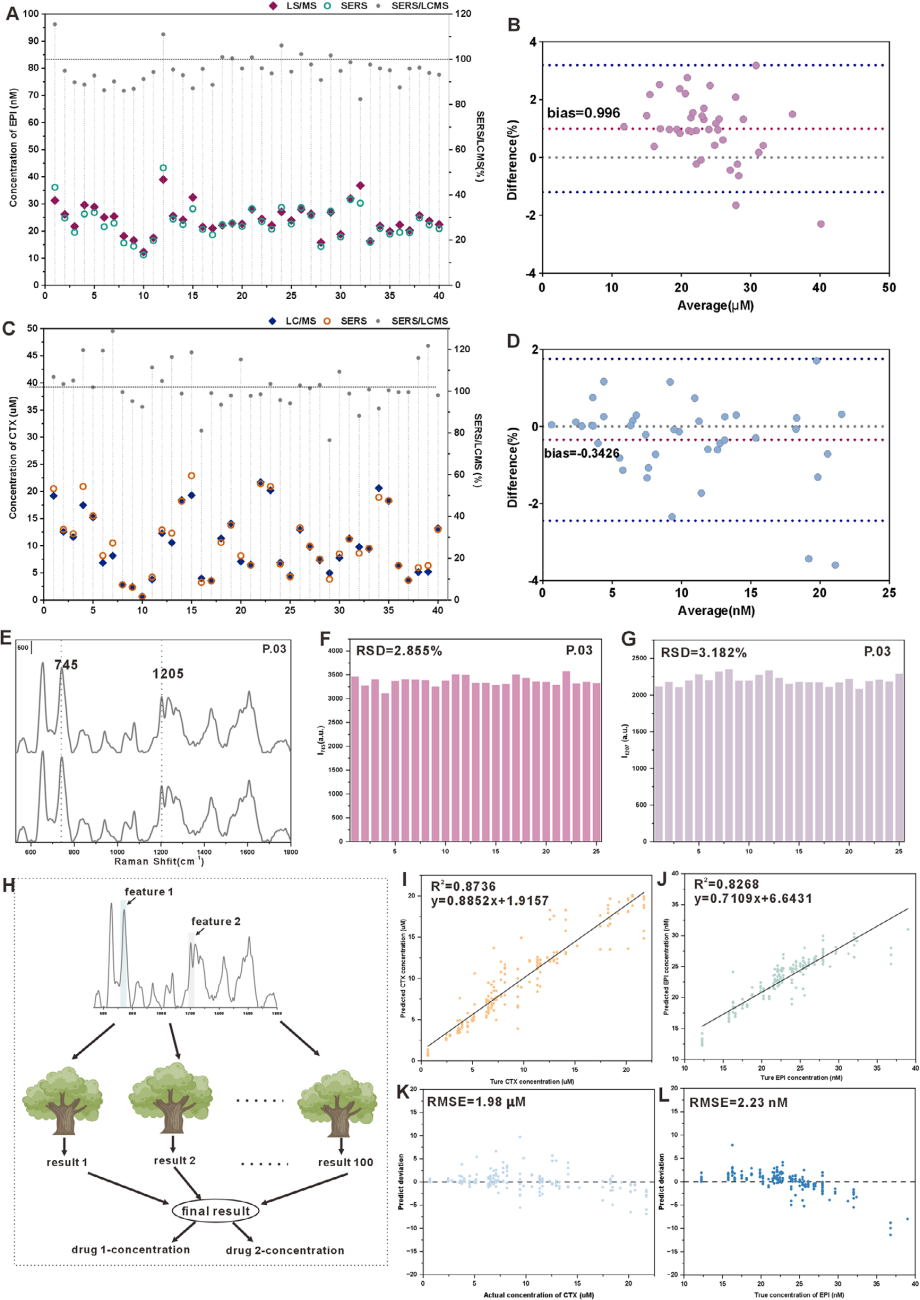
Determination of Drug Concentrations in Patients' Blood Using an mSiO_2_@Ag NPs Self‐Assembled Substrate. (A) Comparison of blood CTX concentrations in patients measured by the mSiO_2_@Ag NPs self‐assembled substrate vs. LC/MS. (B) Bland‐Altman analysis results of two detection methods for EPI drugs. (C) Comparison of blood EPI concentrations in patients measured by the mSiO_2_@Ag NPs self‐assembled substrate vs. LC/MS. (D) Bland‐Altman analysis of two detection methods for CTX drugs. (E) SERS spectrum of serum from Patient No.3. (F) Peak intensity and corresponding RSD at a Raman shift of 745 cm^−1^ for 15 consecutive detections of serum from Patient No.3. (G) Peak intensity and corresponding RSD at a Raman shift of 1205 cm^−1^ for 15 consecutive detections of serum from Patient No.3. (H) Schematic diagram illustrating the data processing workflow using a Random Forest model. (I, J) Linear relationship between RF model‐predicted concentrations and actual concentrations, and RMSE value of CTX. (K, L) Linear relationship between RF model‐predicted concentrations and actual concentrations, and RMSE value of EPI.

To further evaluate architecture‐enabled quantitative SERS in real‐world complex matrices, blood samples collected from patients were analyzed and benchmarked against the gold‐standard LC–MS method. As shown in Figure 5A,C, the concentrations of CTX and EPI determined by the SERS platform were consistent with LC–MS measurements across multiple samples, demonstrating quantitative reliability in real biological matrices. Bland‐Altman analysis revealed a bias of 0.996 for epirubicin and −0.342 for cyclophosphamide, indicating negligible systematic error between the SERS method and LC‐MS reference. All data points fell within the 95% limits of agreement, confirming the reliability of the proposed SERS platform for therapeutic drug monitoring (Figure 5B,D). This consistency confirms that the monolayer architecture maintains chemical specificity and quantitative fidelity even in highly complex biological environments.

Representative SERS spectra acquired from Patient No. 3 (Figure 5E) revealed well‐resolved characteristic Raman features, indicating that analyte‐specific molecular fingerprints could be directly captured from complex serum matrices. Repeated measurements at selected Raman shifts (745 cm^−1^ for CTX and 1205 cm^−1^ for EPI) exhibited excellent signal stability, with RSD values below 10% over 15 consecutive acquisitions (Figure 5F,G). These results further substantiate the robustness and reproducibility of the mesoporous‐shell monolayer architecture under real‐world sampling conditions.

Given the intrinsic spectral complexity of real biological samples and the potential overlap between analyte signals and endogenous biomolecular backgrounds, and predictive performance of four regression models test, a Random Forest (RF) regression model was introduced to enhance quantitative prediction accuracy (Figure 5H and Table S2) [21]. By extracting multidimensional spectral features rather than relying on single‐peak intensities, the RF model effectively captured nonlinear relationships between SERS signatures and analyte concentrations. To verify whether the constructed model relies on authentic Raman signals rather than substrate defects or background noise, permutation importance analysis was conducted in this study. As presented in Figure S10 and Table S3, the key characteristic peaks of CTX are stably concentrated within the range of 740–750 cm^−1^, while the core signature peaks of EPI are distributed at approximately 1200–1210 cm^−1^. The importance scores of other Raman peaks are markedly lower. These findings demonstrate that the model realizes identification based on target characteristic Raman shifts, without interference from random noise or substrate structural defects, thereby endowing the model with favorable physical interpretability. As illustrated in Figure 5I–L, RF‐predicted concentrations of CTX and EPI exhibited strong linear correlations with actual values determined by LC–MS, accompanied by low root mean square error (RMSE). This machine learning–assisted strategy significantly improved the robustness and precision of quantitative SERS in complex biological matrices.

Collectively, these results demonstrate that the integration of a structurally deterministic SERS architecture with data‐driven modeling enables accurate and reproducible molecular quantification directly from complex biological samples. This approach overcomes the limitations of conventional single‐peak calibration in heterogeneous matrices and transforms SERS from a qualitative or semi‐quantitative technique into a decision‐guided quantitative sensing strategy. Validation against LC–MS establishes a critical benchmark for architecture‐enabled quantitative SERS and highlights its potential for feedback‐controlled and decision‐guided molecular readout in real‐world applications.

### 2.6 Architecture‐Enabled Decision‐Guided Sensing for Feedback‐Controlled Functional Validation

**FIGURE 6:**
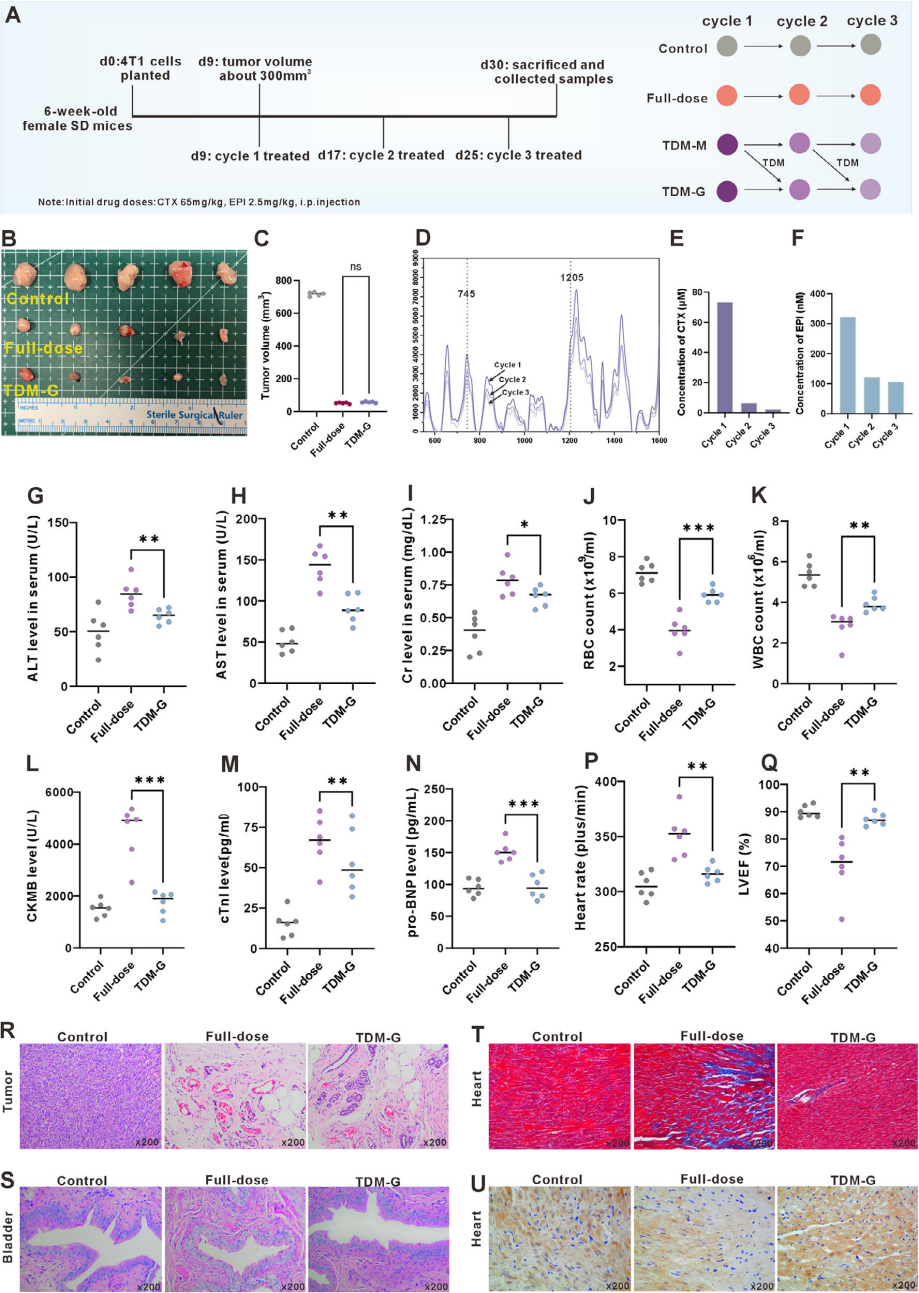
Comparison of Therapeutic Efficacy and Adverse Effects of mice by CTX+EPI Dose Adjustment Guided by SERS Detection. (A) Schematic of the orthotopic breast cancer mouse model and grouping strategy and dose adjustment based on drug concentration monitoring. (B) Tumor images from each group after treatment. (C) Comparison of tumor volumes across groups. (D) The serum SERS detection in TDM‐M mice after each treatment. (E, F) Calculated CTX and EPI concentrations based on SERS results. (G–Q) Comparison of adverse effects: serum levels of (G) ALT, (H) AST, (I) Cr, (J) RBC count, (K) WBC count, (L) CKMB, (M) cTnl, (N) pro‐BNP, (P) heart rate, and (Q) left ventricular ejection fraction% (LVEF%). (R) Representative H&E staining of tumors. (S) Representative PAS staining of bladder. (T) Representative Massion staining of the heart. (U) Representative immunohistochemical staining for CX45 protein of heart.

To validate whether architecture‐enabled quantitative SERS could support feedback‐controlled decision‐making at the functional level, an orthotopic breast cancer mouse model was established and subjected to combination treatment under different dosing strategies (Figure 6A) [22, 23, 24, 25]. Mice were divided into control, full‐dose, SERS‐guided adjustment (SERS‐G), and SERS‐Monitoring (SERS‐M) groups, in which administration was dynamically modulated according to serum concentrations measured by the mesoporous‐shell monolayer SERS architecture. Representative tumor images and quantitative tumor volume analysis revealed that both full‐dose and SERS‐G groups achieved significant growth suppression compared with controls (Figure 6B,C). Notably, growth inhibition in the SERS‐G groups was comparable to that observed in the full‐dose group, indicating that feedback‐controlled adjustment based on quantitative molecular readout preserved functional efficacy. Longitudinal monitoring of tumor volume and body weight further confirmed that SERS‐G adjustment maintained growth suppression while alleviating systemic burden throughout the treatment period (Figure S11).

Serum SERS spectra acquired after each treatment cycle in the SERS‐M group showed consistent and distinguishable molecular features (Figure 6D), enabling quantitative determination of analyte concentrations over successive cycles (Figure 6E,F). These results demonstrate that the architecture enables continuous molecular feedback for real‐time modulation in vivo.

Importantly, while preserving functional efficacy, the decision‐guided strategy markedly reduced systemic burden. Biochemical and hematological analyses revealed significantly lower levels of organ injury markers, including ALT, AST, Cr, CKMB, cTnI, and pro‐BNP, in the SERS‐G compared with the full‐dose group (Figure 6G–N) [26, 27, 28, 29, 30, 31]. In parallel, hematological parameters including RBC and WBC counts were better preserved (Figure 6J,K), indicating improved systemic tolerance [27, 32]. Cardiac functional indices, including heart rate and left ventricular ejection fraction (LVEF), were also significantly improved in the SERS‐G group relative to the full‐dose group (Figure 6P,Q).

Histopathological examinations further corroborated these system‐level observations. H&E staining of tumor tissues confirmed effective cellular damage in both the full‐dose and SERS‐G groups (Figure 6R). In contrast, PAS staining of bladder tissues and Masson staining of cardiac tissues revealed pronounced pathological damage in the full‐dose group, whereas the SERS‐G group exhibited substantially reduced tissue injury (Figure 6S,T). Additional histopathological evidence from bladder tissues further supports the reduced organ damage observed in the SERS‐G group (Figure S12). Immunohistochemical staining for CX45 further demonstrated improved myocardial preservation in mice receiving decision‐guided adjustment (SERS‐G group) (Figure 6U) [33, 34]. Consistent protective effects on cardiac tissue were further confirmed by additional histological analyses, showing reduced myocardial fibrosis and structural damage in the SERS‐G group (Figure S13).

Collectively, these results establish that architecture‐enabled quantitative SERS can be directly integrated into a feedback‐controlled decision loop to optimize functional outcomes in vivo. Rather than relying on predefined dosing schemes, this strategy introduces a paradigm in which molecular exposure is continuously measured and dynamically optimized. This proof‐of‐concept demonstration elevates the mesoporous‐shell monolayer SERS architecture from an analytical sensing tool to a decision‐enabling nanotechnology for feedback‐controlled molecular regulation.

## 3 Conclusion

In this work, we developed a mesoporous‐shell monolayer plasmonic architecture that resolves the long‐standing challenges of instability, poor reproducibility, and limited quantitative reliability in SERS‐based molecular sensing. By integrating controlled molecular transport, chemical protection of photosensitive analytes, and deterministic hotspot geometry, this architecture enables stable, predictable, and chemically faithful SERS responses in complex biological environments. Rather than pursuing maximal electromagnetic enhancement, the design prioritizes quantitative robustness and spectral fidelity, establishing a structural foundation for decision‐guided molecular readout.

Using CTX and EPI as representative small‐molecule analytes, we demonstrated ultrasensitive, reproducible, and multiplexed detection in both standard solutions and serum matrices. Accurate quantification of analyte concentrations in real biological samples was achieved in close agreement with LC–MS through machine learning–assisted spectral modeling. These results show that structurally engineered SERS architectures, when coupled with data‐driven analysis, can provide reliable quantitative information directly from heterogeneous biological systems.

Beyond analytical performance, this study further establishes a feedback‐controlled paradigm in which quantitative SERS is directly linked to functional outcome optimization. In an orthotopic tumor model, decision‐guided adjustment based on SERS‐derived molecular concentrations preserved growth suppression while markedly reducing systemic burden and organ injury. This proof‐of‐concept demonstration elevates SERS from a molecular sensing technique to a decision‐enabling nanotechnology capable of regulating complex biological processes through real‐time molecular feedback.

Collectively, this work bridges nanostructure engineering, quantitative spectroscopy, and feedback‐controlled functional validation, offering a generalizable framework for architecture‐enabled molecular regulation. The concept of combining mesoporous‐shell protection with monolayer hotspot determinism can be extended to other classes of small‐molecule analytes with narrow functional windows. With further integration into portable Raman systems and streamlined sample handling, this strategy holds promise for next‐generation point‐of‐care quantitative sensing and decision‐guided molecular management in complex real‐world environments.

## 4 Methods

### 4.1 Chemicals and Instruments

AgNO_3_, HAuCl_4_, Sodium citrate, and silicon wafers were obtained from Alfa Aesar (Shanghai, China). Crystal Violet (CV), Cyclophosphamide (CTX), and Epirubicin (EPI) were purchased from Aladdin Biochemical Technology Co., Ltd. (Shanghai, China). Acetonitrile (CH3CN), methanol (CH3OH), Cetyltrimethyl Ammonium Bromide (CTAB), NaOH, ascorbic acid, mPEG‐Thiol, isopropanol (3.84 vol.% ammonia (33 wt.%)) and abbr.tetraethyl orthosilicate (TEOS) were obtained from Sigma–Aldrich (USA). The PBS, 0.9% NaCl solution, lactated Ringer's solution, and hematoxylin‐eosin staining kit were all purchased from BIOSHARP (Beijing, China). The hydrophilic‐lipophilic balance (HLB) solid‐phase extraction (SPE) cartridges (60 mg, 3 mL) were obtained from Hunan Bikeman Biotechnology Co., Ltd.

Scanning electron microscopy (SEM) images were acquired using an Auriga focused ion‐beam scanning electron microscope (FIB‐SEM, Zeiss, Germany). Transmission Electron Microscope (TEM) images were obtained by JEM‐F200 (Japan Electronics Co., Ltd, Japan). UV–vis absorption spectra were measured on a Shimadzu UV‐2600 spectrophotometer (Japan). The SERS measurements were conducted on a portable Raman Spectrometer (XS11639, Oceanhood, China). Raman imaging was performed using a Horiba LabRAM HR Evolution confocal Raman spectrometer (HORIBA, France). HE‐stained tissue sections image obteined by an inverted fluorescence microscope (Zeiss, Switzerland). A liquid chromatograph mass spectrometer (LC/MS, Sigma–Aldrich, USA) was used to detect CTX and EPI concentrations.

### 4.2 Standard Solution Preparation

#### 4.2.1 Prepare Gradient Concentrations of CV Solution

First, dissolve 0.408 mg of crystal violet powder in 1 mL of ddH_2_O to obtain a 1 × 10^−3^ M stock solution after complete dissolution. Then, perform serial dilutions from the stock solution to obtain CV solutions with concentrations ranging from 1 × 10^−4^ to 1 × 10^−12^ M.

#### 4.2.2 Gradient Concentrations of CTX and EPI Solution

Dissolve CTX (0.854 mg) or EPI (0.544 mg) powder in 1 mL ddH2O to obtain a 1 × 10^−3 ^M stock solution. Then, perform serial dilutions of the stock solution with ddH_2_O to prepare PTX solutions with concentrations ranging from 1 × 10^−4^ to 1 × 10^−11 ^M.

#### 4.2.3 Gradient Concentrations of CTX and EPI‐Serum Solution

The stock solution was serially diluted with untreated serum to prepare CTX or EPI‐serum solutions at concentrations ranging from 1 × 10^−4^ to 1 × 10^−11 ^M.

### 4.3 Cell Culture and Xenograft Mouse Model

The mice triple‐negative breast cancer cell line 4T1 was purchased from the American Type Culture Collection (ATCC). Cells were cultured in a complete medium consisting of DMEM supplemented with 10% fetal bovine serum and 1% penicillin‐streptomycin antibiotic solution (Gibco, USA), and cultured in a sterile environment at 37°C with 5% CO_2_. Six‐week‐old female [correct strain] mice (Shanghai Model Organisms Center, China) were used for xenograft mouse modeling. The experiment was divided into four groups: control group, full‐dose group, TDM‐monitoring group (TDM‐M), and TDM‐guidance group (TDM‐G), with 5 mice in each group. 4T1 cells in the proliferative phase were mixed 1:1 by volume with Matrigel (1 × 10^5^/mL) and implanted into the fat pad beneath the left eighth mammary nipple of each mouse (80 µL per mouse) to simulate mammary tumor formation. Mouse body weight and tumor volume changes were recorded every three days. On day 9, when the average tumor diameter reached 150 mm^3^, group‐based treatments with PBS and drugs were initiated and administered every 7 days for 3 treatment cycles. day 30, the mice were euthanized, and the tumors were harvested for subsequent experiments.

Treatment regimens for mice in each group were described as follows:

1. Mice in the Control group received intraperitoneal injections of PBS throughout the experiment without any anti‐tumor drug treatment.
2. Mice in the Full‐dose group were treated with full‐dose CTX combined with EPI for the entire treatment course, and the dosage remained unchanged during the experiment.
3. For the TDM‐M (monitoring group) and TDM‐G (guidance group) (formerly denoted as SERS‐M and SERS‐G): After intravenous injection of CTX and EPI in mice, the drugs entered a stable elimination phase at 2 h post‐administration. At 2 h after drug administration, one mouse from the TDM‐M group was sacrificed for blood sampling. SERS measurements were performed to quantify serum drug concentrations, which guided the dosage adjustment for the subsequent treatment cycle of mice in the TDM‐G group.

#### 4.3.1 Dose Adjustment Strategy

The serum concentrations of CTX and EPI were maintained within their respective therapeutic windows (1.5 × 10^−6^–4 × 10^−6^ M for CTX, 8 × 10^−8^–2.5 × 10^−7^ M for EPI) [35, 36]. Preference was given to keeping the blood drug concentration close to the lower limit of the therapeutic window to minimize drug exposure and associated toxic effects. Based on the linear pharmacokinetic assumption (the drugs exhibit linear elimination within the therapeutic concentration range), the dose adjustment formula is as follows: Dose‐new = Dose‐current × (C‐measured/C‐target).

The initial target concentrations were set to 1.5 × 10^−6^ M for CTX and 8 × 10^−8^ M for EPI. If the initially measured blood drug concentration markedly exceeded the upper limit of the therapeutic window, the target concentration for the next administration was adjusted to the corresponding upper limit. In subsequent treatment cycles, the target concentration was gradually tuned downward to maintain drug concentrations close to, but not below, the lower limit of the therapeutic window.

The study was approved by the Animal Experiment Ethics Committee of Hefei Cancer Hospital, Chinese Academy of Sciences [No.DWLLPF‐2025111802].

### 4.4 Human Samples Collection and Pretreatment

40 breast cancer patients receiving CTX+EPI combination chemotherapy in Hefei Cancer Hospital, Chinese Academy of Sciences were selected, and venous blood collection (2 mL per person) was performed 20 h after the completion of CTX+EPI intravenous infusion treatment. Venous blood collection (2 mL per person) was uniformly performed at 8:00 AM from five healthy donors. All patient specimens were divided into two aliquots, one for LC/MS analysis and the other for SERS detection.

200 µL of serum was mixed with 1 mL of methanol and centrifuged at 2000 rpm for 3 min. The entire supernatant was collected and combined with 4 mL of pure water to obtain a preliminary sample. The HLB SPE column was preconditioned by sequentially passing 3 mL of methanol and 3 mL of water through it. The preliminary sample was then loaded onto the column, washed with 2 mL of 2% methanol in water, and finally eluted twice with 200 µL of acetonitrile to obtain the processed sample enriched with target drug molecules. This study obtained informed consent from all patients and their families and was approved by the Medical Ethics Committee of the Hefei Cancer Hospital, Chinese Academy of Sciences[No.PJ‐KY2025‐058].

### 4.5 LC/MS Detection

The internal standard was added to the plasma sample, followed by protein precipitation using acetonitrile. After centrifugation, the supernatant was collected for injection. Separation was performed using an ultra‐high‐performance liquid chromatography system equipped with a C18 column, with a mobile phase consisting of water and methanol, each containing 0.1% formic acid, under a gradient elution program. Detection was carried out using a triple quadrupole mass spectrometer operated in positive ion mode. Quantification was achieved by constructing a standard curve using the internal standard method, and quality control samples were analyzed concurrently to ensure the accuracy and precision of the analytical method.

### 4.6 Fabrication of SERS Substrate

#### 4.6.1 Synthesis and Selection of Nanoparticles

First, silver nanoparticles were synthesized as seeds. The preparation of silver seeds was carried out as follows: In a 250 mL three‐neck round‐bottom flask, 99 mL of ddH_2_O and 1 mL of AgNO_3_ (1 mM) were added, and the mixture was stirred continuously and brought to a boil (100 rpm). After boiling, 3 mL of sodium citrate (1 mM) was added under vigorous stirring (500 rpm). After approximately 3 min, the solution turned white and turbid. Heating and stirring were continued for 1 h to obtain the silver seed solution [37, 38]. In the second step, PEGylation of Ag NPs: The obtained silver seed solution was centrifuged at 18 000 g for 45 min; the supernatant was discarded, and the collected Ag NPs were redispersed in ultra‐filtrated deionized water. Subsequently, under vigorous stirring, the CTAB‐stabilized Ag NPs dispersion was added to an equal volume of an aqueous mPEG‐thiol solution (0.2 mM). The mixture was sonicated for 5 min and then allowed to react for 2 h. Excess mPEG‐thiol molecules were removed by centrifugal filtration at 3000 g for 10 min, and the PEGylated Ag NPs were resuspended in water [37, 38]. The third step involved the growth of a silica shell on the seed surface: The growth solution was heated to 35°C, and 1 mL of the aforementioned seed solution was added. After 15 s, the beaker was removed from the hot plate and left undisturbed for 1 h. This solution was then aged for an additional 12 h at 27°C–30°C, followed by centrifugation twice at 18 000 g for 45 min each time. The collected nanoparticles were redispersed in ultra‐filtrated deionized water. Subsequently, under vigorous stirring, the CTAB‐stabilized AgNPs dispersion was added to an equal volume of an aqueous mPEG‐thiol solution (0.2 mM). The mixture was sonicated for 5 min and then allowed to react for 2 h. Excess mPEG‐thiol molecules were removed by centrifugal filtration at 3000 g for 10 min, and the PEGylated AgNPs were resuspended in water. In the fourth step, a mesoporous silica shell was grown on the PEGylated AgNPs using a CTAB‐templated modified Stöber method [37, 38]. First, 0.2 mL of CTAB (10 mM) was added to 1.2 mL of the PEGylated AgNPs suspension under gentle stirring for 30 min to allow CTAB adsorption as a mesopore template. The mixture was then added with vigorous stirring to 1.8 mL of isopropanol, followed by slow addition of an ammonia solution in isopropanol (3.84 vol.% ammonia (33 wt.%)) until the solution reached pH = 11. Subsequently, 0.030 mL of a TEOS solution (100 mM) in isopropanol was added under gentle stirring. The reaction mixture was left undisturbed for 2 h to allow silica condensation around the CTAB micelles. The resulting product was collected by centrifugation (18 000 g, 30 min) and washed twice with ethanol. Finally, to remove the CTAB template and generate mesopores, a solvent extraction method was adopted [6, 7, 8]. The as‐synthesized particles were dispersed in 10 mL of an ethanol solution containing 0.5 wt.% NH_4_NO_3_, and the mixture was stirred at 60°C for 1 h. This extraction process was repeated twice. Finally, the CTAB‐removed mesoporous silica‐coated Ag nanoparticles (mSiO_2_@Ag NPs) were washed twice with ethanol and redispersed in deionized water for further use [39, 40].

#### 4.6.2 Air–Liquid Interface Self‐Assembly

First, ultrapure water was injected into a clean Langmuir trough as the subphase. Subsequently, mSiO_2_@Ag NPs were uniformly dispersed in methanol and then slowly dropped and spread onto the subphase surface. After complete evaporation of the solvent, the nanoparticles formed a loosely distributed initial monolayer at the air–liquid interface. Next, the monolayer was subjected to slow isothermal compression using a movable barrier until a preset target surface pressure was reached, thereby forming a densely packed and well‑ordered solid nanoparticle monolayer. Finally, the ordered monolayer was completely transferred onto a pretreated silicon wafer substrate via vertical lifting, and the resulting sample was reserved for subsequent SERS detection and analysis.

### 4.7 SERS Measurements

The substrates prepared by gas–liquid interface self‐assembly using mesoporous silica‐coated silver nanoparticles (mSiO_2_@Ag NPs) were used for surface‐enhanced Raman spectroscopy (SERS) measurements in a wet state. A 5 µL aliquot of the sample solution was dropped onto the substrate, forming a droplet with a diameter of approximately 2–3 mm. No special drying treatment was applied after droplet deposition. SERS detection was performed 5 s after sample loading. The SERS measurements were conducted on a portable Raman Spectrometer (XS11639, Oceanhood, China). A 785 nm laser with a maximum laser power of 80 mW and a time of 50–100 ms was used for all the SERS measurements. A 785 nm laser with a laser power of 150 mW and a time of 100 ms was used for all the Raman measurements.

### 4.8 Raman Enhancement Factor Calculation

Raman enhancement factor (EF) was calculated as follows:

(1) $$\mathrm{EF}=\left(\frac{I_{\mathrm{SERS}}}{N_{\mathrm{SERS}}}\right)/\left(\frac{I_{\mathrm{Raman}}}{N_{\mathrm{Raman}}}\right)$$

*I_SERS_* means signal intensity measured under SERS conditions (1618 cm^−1^), *I_Raman_* means signal intensity measured under Raman conditions (1618 cm^−1^), *N_SERS_* means the number of molecules to be measured in the laser‐irradiated region, and *N_Raman_* means the number of molecules to be measured adsorbed under the laser‐irradiated region.

### 4.9 Machine Learning

To establish the quantitative relationship between SERS spectral intensity and target analyte concentration, this study compared the predictive performance of four regression models. The dataset was divided into a training set, validation set, and test set at a ratio of 8:1:1. To evaluate the stability and generalization ability of the models, we compared the performance differences of each model on the validation set and test set. The specific results are shown in Table S2. It can be seen that the random forest regression model exhibited the best predictive performance on both the validation set and the test set, with a difference in R^2^ between the validation set and test set of only 0.0007, indicating excellent generalization ability and no overfitting. Therefore, the random forest model was selected for prediction.

Given the significant spatial heterogeneity in the SERS system (signal differences across different substrates can reach up to ∼1000%), randomly splitting the spectral data would likely cause spectra from the same substrate to appear in both training and validation sets, leading to data leakage and overestimation of model performance.

Therefore, we adopted a Group K‑Fold cross‑validation strategy:

1. Each folder corresponds to an independent substrate (containing multiple spectra acquired on that substrate).
2. During cross‑validation, the folder is used as the group unit for splitting.
3. All spectra from the same substrate are assigned exclusively to either the training set or the validation set.

Dataset Directory Structure Is as Follows:

│── 40 Samples/

│ │── Concentration1(substrate1)/

│ │ │── Xxx.csv

│ │ │── Xxx.csv

│ │ │── ….

│ │── Concentration 2(substrate2)/

│ │── …

This prevents data leakage and ensures that the model is evaluated on unseen spatial domains. The 5‑fold cross‑validation results are shown in Table S3. The overall average cross‑validation results are: R^2^ = 0.8551 for Drug1, and R^2^ = 0.8190 for Drug 2. Performance is stable across folds, with no obvious performance collapse, indicating good cross‑substrate generalization ability rather than reliance on specific substrate characteristics.

At the same time, we performed external validation using an independent batch (substrate‑level split): 80% of the substrates were used for training and 20% as a completely independent test set. No spectral data from the test groups were used during training or model selection. External validation results: for Drug 1, R^2^ = 0.8426; for Drug 2, R^2^ = 0.8039. Key analysis shows that the external R^2^ of Drug1 is close to the cross‑validation mean, indicating stable generalization without significant overfitting. The external R^2^ of Drug 2 is also consistent with the CV results, demonstrating that the model captures a stable signal rather than random noise.

To verify whether the model relies on genuine Raman signals rather than substrate defects or noise, we performed permutation importance analysis. The results (as shown in the figure) indicate that the most important features for Drug 1 are consistently in the characteristic peak region around 740–750 cm^−1^, and for Drug 2 around 1200–1210 cm^−1^, while the importance of other peaks is significantly lower. The model primarily relies on the target Raman shift rather than random noise or substrate structural defects; the model has clear physical interpretability.

To further reduce the risk of overfitting, we also introduced the following strategies:

1. Adaptive peak detection: peak prominence = noise level × coefficient, peak height threshold = spectral maximum intensity × ratio, improving robustness under different spectral conditions.
2. Outlier removal: Z‑score method was used to eliminate anomalous spectra, avoiding extreme values interfering with model training.
3. Hyperparameter tuning within cross‑validation: GridSearchCV was used to optimize parameters on the training set.

The maximum peak intensity within the characteristic Raman bands was extracted as the feature. Z‐score outlier removal (threshold: 2.5σ) was applied per concentration. The dataset was split into training, validation, and test sets (8:1:1) via stratified sampling to ensure balanced concentration distribution. The feature was standardized using the training set's mean (µ) and standard deviation (σ). Four regression models—Linear, Ridge, Random Forest (RF), and Support Vector Regression—were compared. RF, configured with 100 trees utilizing bootstrap aggregation and random feature selection, demonstrated superior performance and was selected as the final model. Models were trained on the training set, with hyperparameters tuned on the validation set. Final evaluation on the independent test set employed R^2^ and RMSE. All analysis was performed in Python using scikit‐learn, pandas, and numpy. Results were visualized with matplotlib/seaborn, presenting predicted vs. actual concentration plots with a reference line (*y* = *x*) and model performance metrics.

### 4.10 Statistical Analysis

Calculations and graphing were performed using SPSS (version 22.0), Origin (version 2025), Prism (version 7.0), and CorelDRAW (version 2024). All experiments were repeated at least 3 times (*n* ≥ 3). A paired *t*‐test was used for comparisons between two groups, Bland‐Altman analysis was used to identify systematic biases, and analysis of variance was applied for comparisons among multiple groups. *p* < 0.05 was considered statistically significant. Note: ^*^ *p* < 0.05, ^**^ *p* < 0.01, ^***^ *p* < 0.001, ^****^ *p* < 0.0001.

## Author Contributions

G.H., Y.W., and Y.F. contributed equally to this work. G.H., Y.W., Y.F., and W.Z. conceived and designed the research. G.H. and Y.W. developed the mesoporous‐shell monolayer SERS platform and performed nanomaterial synthesis and structural characterization. Y.F. and J.S. carried out SERS measurements and quantitative analysis of chemotherapeutic drugs. S.H., M.Z., and Y.Y. assisted with data acquisition, sample preparation, and statistical analysis. J.S. and J.W. contributed to biological experiments and serum sample analysis. W.Z. provided critical input on experimental strategy, nanomaterial fabrication, and mechanistic interpretation, and finalized the manuscript. H.W. supervised the overall project and provided guidance on experimental design and data interpretation. All authors discussed the results and contributed to manuscript writing and revision. All authors approved the final version of the manuscript.

## Trial Registration

Not applicable. The clinical component of this study involve analysis of patient‐derived blood samples for analytical method validation and did not involve prospective assignment of human participants to a health‐related intervention.

## Code Availability Statement

All the code used for AI algorithms is available from GitHub.

## Conflicts of Interest

The authors declare no conflicts of interest.

## Supporting information




**Supporting File**: advs77299‐sup‐0001‐SuppMat.docx.

## Data Availability

The data that support the findings of this study are available from the corresponding author upon reasonable request.
