# Supplementary material for: Mesoporous‐Shell Monolayer Plasmonic Architecture Enables Quantitative and Decision‐Guided SERS
Source: Adv Sci (Weinh). 2026 Aug 24;13(60):e77299. doi: 10.1002/advs.77299 (PMC13502281; doi:10.1002/advs.77299)
Supplement: Supplementary file 1 [file ADVS-13-e77299-s001.docx]

*Supplementary Material*

**Mesoporous-Shell Monolayer Plasmonic Architecture Enables Quantitative and Decision-Guided SERS**

*Guangyao Huang^1, #^, Ying Wang^1, 2, #^, Yue Fang^1, #^, Jingyuan Shao^1, 2^, Sensen Hao^1^, Mancun Zhao^1^, Yuan Yao^1^, Junping Wang^1^, Wei Zhou^3,^ *, Hongzhi Wang^1, 2,^ **

^1^ Hefei Cancer Hospital of CAS, Institute of Health and Medical Technology, Hefei Institutes of Physical Science, Chinese Academy of Sciences (CAS), No. 350, Shushan Hu Road, Hefei , Anhui , China;

^2^ University of Science and Technology of China, Hefei, China;

^3^ Beijing Institute of Radiation Medicine, 27 Taiping Road, Beijing 100850, China.

**^#^Authors contributed equally to this work.**

*** Corresponding Authors:**

Wei Zhou, Associate Professor, E-mail: [zhouweisyl802@163.com](mailto:zhouweisyl802@163.com)

Hongzhi Wang, Professor, E-mail: [wanghz@hfcas.ac.cn](mailto:wanghz@hfcas.ac.cn)

**Supplement Figure 1.** TEM images of the bare Ag nanoparticles

**Supplement Figure 2. Peak intensities and corresponding RSD values for repeated measurements of high-concentration drug solutions using the mSiO₂@Ag NPs self-assembled substrate. A.** Peak intensity and RSD for 15 consecutive measurements of 1×10⁻⁵ M CTX. **B.** Peak intensity and RSD for 15 consecutive measurements of 1×10⁻⁶ M EPI.

**Supplement Figure 3. Measurement results at five random different points on the mSiO₂@Ag NPs self-assembled substrate. A–E.** Measurement results of random points 1–5. **F.** Comparison of peak intensities for the five random points.

**Supplement Figure 4. Measurement results of five mSiO₂@Ag NPs self-assembled substrates from the same batch. A–E.** Measurement results of substrates 1–5. **F.** Comparison of peak intensities among the five substrates.

**Supplement Figure 5. Measurement results of mSiO₂@Ag NPs self-assembled substrates from five different batches. A–E.** Measurement results of substrates from batches 1–5. **F.** Comparison of peak intensities among substrates of the five batches.

**Supplement Figure 6. Calibration curve of CV concentration versus peak intensity.**

**Supplement Figure 7. Detection of Carboplatin (Cb) Using an mSiO₂@Ag NPs Self-Assembled Substrate. A.** Comparison of conventional Raman and SERS spectra of carboplatin for identifying characteristic peaks. **B.** Detection of carboplatin at different concentrations using the mSiO₂@Ag NPs self-assembled substrate.

**Supplement Figure 8. Detection of Dacarbazine (DTIC) Using an mSiO₂@Ag NPs Self-Assembled Substrate. A.** Comparison of conventional Raman and SERS spectra of dacarbazine for identifying characteristic peaks. **B.** Detection of dacarbazine at different concentrations using the mSiO₂@Ag NPs self-assembled substrate.

**Supplement Figure 9. SERS detection of serum samples pretreated with HLB using substrates assembled with different particles.**

**Supplement Figure 10. Feature Importance of CTX and EPI drugs. A and B.** Analysis of Stable Peak Importance for CTX and EPI Key Features.

**Supplement Figure 11. Body weight and tumor volume change curves for mice in each group. A.** Body weight change curves for each group of mice. **B.** Tumor volume change curves for each group of mice.

**Supplement Figure 12. representative H&E staining of bladders.**

**Supplement Figure 13. representative H&E staining of hearts.**

**Supplement Table 1.** Tentative assignments and qualitative changes in the Raman bands before and after serum pretreatment.

| **Raman shift (cm^-1^)** | **Tentative assignment** | **Before pretreatment** | **After pretreatment** | **Overall classification** | **Supporting data** |
| --- | --- | --- | --- | --- | --- |
| 700–730 | Overlapping nucleobase- and phospholipid-associated vibrations | Strong and clearly detectable as part of the serum background | Markedly attenuated; weak residual contributions may remain | Suppressed | Figure 4B |
| ~745 | CTX marker band, predominantly associated with a C–Cl vibration | Partially obscured or not reliably resolved against the untreated serum background, particularly at low CTX concentrations | Clearly detectable in processed CTX-containing samples | Retained and unmasked | Figures 3A, 4C, and 4I–K |
| 830–850 | Tyrosine-associated ring vibrations | Clearly detectable in untreated serum | Markedly attenuated after protein precipitation and HLB extraction | Suppressed | Figure 4B |
| ~1000 | Phenylalanine ring-breathing vibration and associated protein contributions | Strong and clearly detectable | Markedly attenuated | Suppressed | Figure 4B |
| 1120–1150 | Protein- or lipid-associated C–C and C–N stretching vibrations | Clearly detectable as part of the broad serum background | Attenuated; weak overlapping signals may remain | Suppressed | Figure 4B |
| ~1205 | EPI marker band associated with an anthracycline ring vibration | Partially obscured or not reliably resolved against the untreated serum background, particularly at low EPI concentrations | Clearly detectable in processed EPI-containing samples | Retained and unmasked | Figures 3E, 4F, and 4I–K |
| 1240–1300 | Amide III and lipid-associated CH_2_ twisting or wagging modes | Strong and broad | Markedly attenuated | Suppressed | Figure 4B |
| 1370–1450 | CH_2_ and CH_3_ deformation modes from proteins and lipids | Strong and broad | Markedly attenuated; weak residual background may persist | Suppressed | Figure 4B |

The assignments of the serum-derived bands are tentative because the observed spectral regions contain overlapping contributions from multiple serum constituents. “Suppressed” indicates a substantial reduction in intensity rather than necessarily complete disappearance. The CTX and EPI marker bands were evaluated in the corresponding drug-containing samples.

**Supplement Table 2. Performance comparison of different regression models**

| Model | Validation Set R² | Test Set R² |
| --- | --- | --- |
| Linear Regression | 0.9921 | 0.9924 |
| Ridge Regression | 0.9923 | 0.9926 |
| Random Forest | 0.9971 | 0.9978 |
| Support Vector Regression | 0.9534 | 0.9523 |

**Supplement Table 3. Cross-validation and external validation results**

|  | Drug 1 | | | Drug 2 | | |
| --- | --- | --- | --- | --- | --- | --- |
|  | R² | RMSE | MAE | R² | RMSE | MAE |
| Overall CV results | 0.8551 ± 0.1260 | 0.1411 ± 0.0645 | 0.1037 ± 0.0343 | 0.8190± 0.1277 | 0.0384 ± 0.0138 | 0.0273 ± 0.0088 |
| External Test | 0.8426 | 0.1020 | 0.0853 | 0.8039 | 0.0247 | 0.0164 |
